# All‐Optic Logical Operations Based on the Visible‐Near Infrared Bipolar Optical Response

**DOI:** 10.1002/advs.202404336

**Published:** 2024-07-23

**Authors:** Jie You, Zhao Han, Ningning Zhang, Qiancui Zhang, Yichi Zhang, Yang Liu, Yang Li, Jinping Ao, Zuimin Jiang, Zhenyang Zhong, Hui Guo, Huiyong Hu, Liming Wang, Zhangming Zhu

**Affiliations:** ^1^ Key Laboratory of Analog Integrated Circuits and Systems (Ministry of Education) School of Integrated Circuits Xidian University Xi'an 710071 China; ^2^ School of Integrated Circuits Jiangnan University Wuxi Jiangsu 214000 China; ^3^ State Key Laboratory of Surface Physics Department of Physics Fudan University Shanghai 200433 China

**Keywords:** all‐optic logical, bipolar optical response, reduction transistor number

## Abstract

The burgeoning need for extensive data processing has sparked enthusiasm for the development of a novel optical logic gate platform. In this study, junction field‐effect phototransistors based on molybdenum disulfide/Germanium (MoS_2_/Ge) heterojunctions are constructed as optical logic units. This device demonstrates a positive photoresponse that is attributed to the photoconductivity effect occurring upon irradiation with visible (Vis) light. Under the illumination of near‐infrared (NIR) optics with wavelengths within the communication band, the device shows a negative photoresponse, which is associated with the interlayer Coulomb interactions. The current state of the device can be effectively modulated as different logical states by precisely tuning the wavelength and power density of the optical. Within a 3 × 3 MoS_2_/Ge phototransistor array, five essentially all‐optical logic gates (“AND,” “OR,” “NAND,” “NOT,” and “NOR”) can be achieved in every signal unit. Furthermore, three complex all‐optical logical operations are demonstrated by integrating two MoS_2_/Ge phototransistors in series. Compared to electronic designs, these all‐optical logic devices offer a significant reduction in transistor number, with savings of 50–94% when implementing the above‐mentioned functions. These results present opportunities for the development of photonic chips with low power consumption, high fidelity, and large volumes.

## Graphical abstract

Ge/MoS_2_ JFET demonstrates a remarkable bipolar optical response in the visible and infrared spectrum, achieving various essentially and complex all‐optical logic operations. Compared with traditional electronic circuits, all‐optical devices reduce the number of transistors by more than 50%. Capitalizing on these distinctive characteristics, this device holds immense promise for innovative all‐optical image processing.

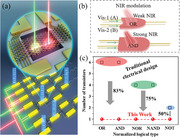

## 1 Introduction

In electronic circuits, logical states are defined as the electrical states within circuit components, which are typically represented by “0” and “1” to indicate the results of logical operations.^[^ ^1^ ^]^ These logical states are realized through the engineering of fundamental device structures, such as p‐n diodes or metal–oxide–semiconductor field‐effect transistors.^[^ ^2^ ^]^ Various types of logical gates, such as “AND,” “OR,” “NAND,” “NOT,” and “NOR”, are used for sophisticated computing tasks by interconnecting individual logical units.^[^ ^3^ ^]^ Despite the extensive development of electrical logic operations, the continuous reduction of semiconductor process nodes poses challenges such as increased power consumption, decreased stability, and significant thermal effects during electrical signal processing, hindering the construction of high‐performance chips.^[^ ^4^ ^]^ To address these challenges, some efforts have been made to improve the circuit performance at low‐nanometer nodes through the development of new materials and the optimization of design structures.^[^ ^5^ ^]^ However, reliance on electrons as signal carriers at the nanoscale is subject to the effects of quantum tunneling, which presents a formidable obstacle to resolving the aforementioned issues.^[^ ^6^ ^]^ In contrast, photons offer distinct advantages over electrons, including low power consumption, minimal thermal limitations, high speed, and the ability to facilitate broadband communication.^[^ ^7^ ^]^ Using photons for information transmission and processing offers significant potential for overcoming existing technological bottlenecks of electronic systems and can pave the way for promising advancements in the field of logical devices.

Due to the outstanding properties and wide application scenarios of all‐optical logical operations, it is imperative to use optical devices to achieve future computing trends.^[^ ^8^ ^]^ In recent years, several typical approaches with different schemes have been proposed to design all‐optical logical gates, such as spatial encoding of light fields, nonlinear photonic crystals, highly nonlinear fibers, two‐photon absorption, and semiconductor optical amplifiers.^[^ ^9^ ^]^ In these approaches, where optical signals serve as both input and output, compatibility with existing integrated circuit processes become a significant challenge. Furthermore, limitations such as large volume, complex design, involvement of nonlinear media, and difficulties in controlling relative phase differences of input signals have hindered the commercialization prospects of photonic computing devices.^[^ ^10^ ^]^

To address these issues, the method of optical input and electrical output has gained widespread attention.^[^ ^11^ ^]^ A light‐modulated organic semiconductor device with a back‐to‐back pn junction was proposed, which offers valuable insights for the construction of all‐optical logic devices.^[^ ^12^ ^]^ The typical device features a band alignment p^+^‐i‐n‐p‐p^+^ diode structure, effectively demonstrating the feasibility of fundamental all‐optical logic operations. However, despite these advancements, several notable challenges remain and impede the commercialization of all‐optical logical gates. First, the back‐to‐back pn junction structure necessitates the utilization of at least five band‐matched semiconductor materials, leading to complex design and manufacturing processes. Second, the reported all‐optical logic primarily operates within the Vis light range, rather than the NIR communication light that is compatible with silicon photonics processes. Consequently, designing on‐chip photonic logic devices that are compatible with existing silicon photonics processes becomes challenging. Third, there are few studies on multidevice cooperative signal processing to implement complex logic functions, which is crucial for the development of large‐scale integrated circuits.

Leveraging the abovementioned all‐optic logical design concept, the integration of 2D and 3D materials has the potential to overcome these challenges.^[^ ^13^ ^]^ By selecting an appropriate material system, such as Si or Ge, the resulting device can be suitable for the development of on‐chip photonic logic chips that are compatible with silicon photonics processes.^[^ ^14^ ^]^ Moreover, the 2D/3D integration structure offers additional advantages, including a high absorption coefficient and high integration density.^[^ ^15^ ^]^ These features facilitate the design and implementation of complex logic functionalities through multidevice cooperation.^[^ ^16^ ^]^ Consequently, this method offers significant promise as a candidate for realizing all‐optical logical gates and potentially replacing existing technologies.

In this work, a MoS_2_/Ge heterojunction field‐effect transistor (JFET) is fabricated. Based on the photoconduction in the channel and interlayer Coulomb interactions at the heterojunction interface under Vis and NIR optics, the device exhibits a wavelength‐dependent bipolar optic response characteristic. This beneficial performance can be strategically used to implement varied optical logic. Five representative basic logical gates (“AND,” “OR,” “NAND,” “NOT,” and “NOR”) are demonstrated on a single MoS_2_/Ge JFET unit within a 3 × 3 array, and the different logical gates can be transformed by changing the wavelength and power density of the modulation and input optics. Based on the abovementioned characteristics of the device, various of simple image processing can be completed by encoding the image into a binary optical input signal. Moreover, complex all‐optical logical operations (e.g., “*Y* = *A*·*B +* $\bar{C}$,” “*Y* = $\bar{A}$·$\bar{B}+\bar{B}$·*C* + $\bar{A}$·$\bar{C}$,” and “*Y* = $\bar{A}$·$\bar{B}$ + *C*”) can be achieved by integrating two MoS_2_/Ge JFETs in series. The transistor number of all‐optical logic operations is reduced by more than half as compared to electronic logical devices when implementing basic and complex logical operations. These results offer significant advantages in terms of spatial efficiency and cost‐effectiveness compared to conventional electronic logic circuits. Furthermore, the devices hold immense potential for advancing the development of high‐speed, high‐throughput, and intricate data processing in optical computing in future applications.

## 2 Results

**Figure 1:**
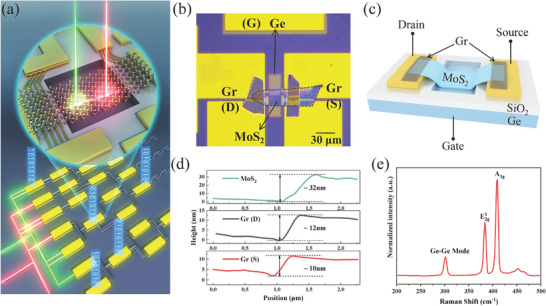
Device structure and materials characterization. a) Schematic diagram of an all‐optical logical circuit. Inset: the amplified structure of a single device in this circuit. Optical microscope image b) and schematic illustration c) of the MoS_2_/Ge JFET structure. d) Measured thicknesses of MoS_2_, drain, and source Gr from AFM results. e) Raman spectra of the MoS_2_/Ge JFET.

**Figure** **1**a depicts a schematic diagram of an all‐optical logic circuit, where optical signals serve as inputs and electrical signals serve as outputs. Data transmission between the devices occurs through electrical signals. Figure 1b,c depicts the optical image and 3D schematic illustration, respectively, of the n‐MoS_2_/p‐Ge JFET structure. Here, MoS_2_ and Ge serve as absorption materials of Vis and NIR optics. Additionally, Ge is used as the gate to adjust the carrier transport characteristics in the MoS_2_ channel, and graphite (Gr) electrodes significantly reduce the contact resistance and improve the carrier collection efficiency. Figure 1d displays the atomic force microscopy (AFM) results of the 2D materials, with thicknesses of MoS_2_, drain Gr and source Gr are ≈32, 12, and 10 nm, respectively. Ge and MoS_2_ are clearly identified by the Raman spectra, as shown in Figure 1e, with Ge–Ge (≈301 cm^−1^), E_2g_ (≈381 cm^−1^), and A_1g_ (≈407 cm^−1^) peaks matching those of Ge and multilayer MoS_2_ reported prevoiusly.^[^ ^17^ ^]^

**Figure 2:**
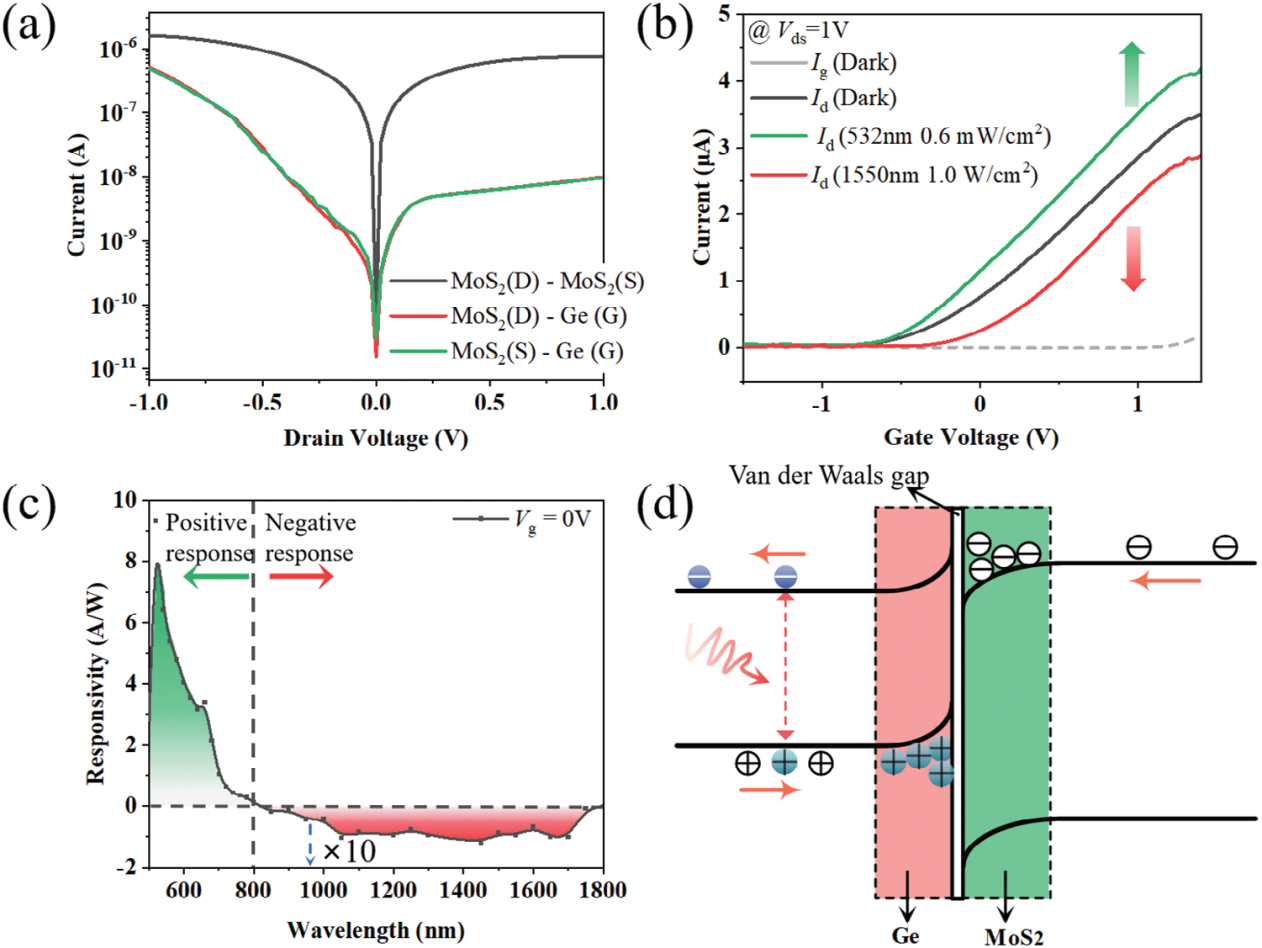
Electrical and photoelectric properties. a) Output characteristics of the MoS_2_ channel and MoS_2_‐Ge heterojunction. b) Transfer curves of the structure at a 1 V drain voltage bias under dark and optical illumination (the power densities at 532 and 1550 nm are 0.6 mW cm^−2^ and 1.0 W cm^−2^, respectively). c) Bipolar photoresponse spectra of the JFET as a function of wavelength in the Vis and NIR bands from 500 to 1800 nm. d) Energy band diagram of the heterojunction in the JFET under NIR optic illumination.

In **Figure** **2**a, the black line depicts the output characteristic of the MoS_2_ JFET, where the currents at the drain and source of the MoS_2_ channel are basically consistent. The red and green lines in Figure 2a represent the heterojunction characteristics of Ge/MoS_2_(drain) and Ge/MoS_2_(source), respectively. The ON/OFF current ratio reaches ≈10^2^, indicating a typical heterojunction characteristic between n‐MoS_2_ and p‐Ge. Figure 2b and Figure S1 (Supporting Information) present the linear‐scaled and log‐scaled transfer curves under dark and optical illumination, respectively. Within the range of gate voltages (−1.5 to 1.5 V), the gate leakage current (*I*_g_) is negligible. This JFET device exhibits both positive and negative photocurrent characteristics under illumination at 532 and 1550 nm, respectively, which enables the realization of all‐optical logic operations. Figure S2a,b (Supporting Information) illustrates the time‐dependent positive and negative response of the device under illumination at 532  and 1550 nm, respectively. It is observed that the photocurrent increases with an increase in the optical power. The operation optics and device photocurrent functioned as logic input and output states, respectively. Various logic functions are realized by designing combinations of different wavelengths and optics intensities. Figure S3a,b (Supporting Information) illustrates the device's dynamic optical response and light source power over a wide spectrum, respectively. Figure 2c shows the bipolar photoresponse spectrum of the JFET in the range of 500–1800 nm, with a net‐zero photocurrent occurring at ≈800 nm. The MoS_2_ layer exhibits a positive photoresponse in the Vis range (500–800 nm), and the negative photoresponse beyond 800 nm is mainly attributed to absorption by the Ge material. The net‐zero photocurrent at 800 nm is a result of competing positive and negative photoresponses.

To quantitatively investigate the energy band alignments in the MoS_2_/Ge JFET, kelvin probe force microscopy (KPFM) was performed to measure the surface potential across the interface. Figure S4a (Supporting Information) displays a KPFM image of the Ge/MoS_2_ heterojunction, and the potential result along the white line in Figure S4a (Supporting Information) is presented in Figure S4b (Supporting Information). The contact potential difference (*V*_CPD_), which is defined as the difference in work function between the probe and material, can be calculated as *V*_CPD_ = (*W*_Probe_ – *W*_material_)/q, where *W*_Probe_ and *W*_material_ represent the work function of the probe and material, respectively, and q is the elementary electronic charge.^[^ ^18^ ^]^ Notably, a 0.24 eV potential difference (Δ*V*_CPD_) between Ge and MoS_2_ is revealed and shown in Figure S4b (Supporting Information). Based on this result, a schematic energy band diagram before contact is depicted in Figure S4c (Supporting Information), and the offset between Ge valence band and MoS_2_ conduction band (E_v(Ge)_‐E_c(MoS2)_) is 0.49 eV. Due to the work function mismatch of Ge and MoS_2_, the energy band of MoS_2_ bends downward and Ge bends upward after contact, producing an internal electric field from Ge to MoS_2_ (Figure S4d, Supporting Information). There is a large difference between the conduction band (ΔE_c_ = 0.27 eV) and valence band (ΔE_v_ = 0.81 eV) of Ge and MoS_2_.^[^ ^19^ ^]^ As a result, the electrons and holes in the space charge region are confined to the Ge/MoS_2_ interface close to the sides of MoS_2_ and Ge, respectively. Due to the subnanometer feature of van der Waals gaps at the MoS_2_/Ge interface, Coulomb interactions are produced between confined electrons and holes.^[^ ^20^ ^]^

Figure 2d displays the energy band diagram of the MoS_2_/Ge heterojunction under illumination of the NIR optic. Due to the limited band gap of MoS_2_ (E_g_ = 1.2 eV), the absorption of the NIR optic is mainly concentrated in Ge. The colored spheres for Ge represent photogenerated electrons and holes, which move in the direction indicated by the arrows. Subsequently, a large number of holes accumulate near the Ge side of the MoS_2_/Ge interface, attracting more electrons in the MoS_2_ channel to the interface under the influence of Coulomb interactions. Consequently, the density of effective carriers in MoS_2_ is reduced, resulting in a negative photoresponse under NIR illumination.^[^ ^21^ ^]^ Currently, there are numerous literatures that have reported that in the specific band structure TMD van der Waals heterojunction, the separated electrons and holes in different materials have Coulomb interactions.^[^ ^22^ ^]^ To further confirm the Coulomb interactions theory, photoluminescence spectrum under 532 nm excitation at 77 K is measured (Figure S5, Supporting Information), and two energy peaks of Ge (blue dashed box, 0.7 eV) and offset of E_v(Ge)_‐E_c(MoS2)_ (red dashed box, 0.48 eV) were observed. This property is considered to be the main reason for the negative response in the NIR band. Under Vis illumination, MoS_2_ is the primary absorption layer, and the increase of effective carrier density in the MoS_2_ channel produces a positive photocurrent.

**Figure 3:**
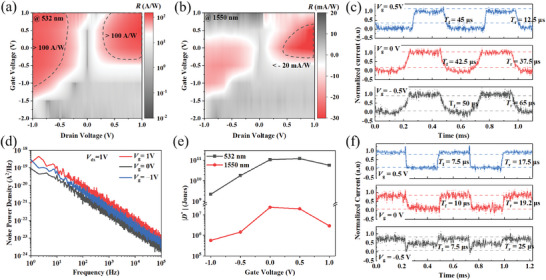
Photoelectric parameter characterization. Responsivity contour maps (*V*_g_ vs *V*_d_) of the MoS_2_/Ge JFET under 532 nm a) and 1550 nm b) optic illumination, respectively. d) Noise power spectra of MoS_2_/Ge JFET under different gate biases. e) Absolute values of detectivity as a function of gate bias upon illumination at 532 and 1550 nm.Temporal response of the JFET at −0.5, 0, and 0.5 V gate voltage biases under 532 c) and 1550 nm f) illumination.

To obtain the optimal working parameter conditions of all‐optical logical operation, the *V*_g_ and *V*_d_ dependent responsivity (*R*), detectivity (*D* ^*^), and response time (*T*_f_ and *T*_r_) characteristics are analyzed. *R* and *D* ^*^ indicate the photoelectric conversion capability and the minimum detection optical power. *R* can be calculated by *R* = (*I*_Light_ − *I*_Dark_)/*P*_eff_,^[^ ^23^ ^]^ where *I*_Light_ and *I*_Dark_ are the currents under optical illumination and in the dark, respectively, *P*_eff_ is the effective incident power density. **Figure** **3**a,b demonstrates the *R* contour maps of the JFET device under 532 and 1550 nm illumination, respectively, with this parameter being strongly related to *V*_g_ and *V*_d_.^[^ ^24^ ^]^ As shown in Figure 3a,b, regions with *R* > 100 A/W and *R <* −20 mA/W can be observed in the dashed‐line regions. The positive photoresponse in the Vis optic and negative photoresponse in the NIR are the basis for implementing all‐optical logical operation. The low *R* under 1550 nm illumination can be attributed to two primary factors: Ge generates a large number of photocarriers upon NIR illumination, indirectly affecting the transport state of channel carriers under the influence Coulomb interaction; Additionally, during *R* testing, the device is illuminated with free space light through fiber coupling. A larger spot area reduces the effective optical power on the device, resulting in a low calculated responsivity. Integrating the device on‐chip and transmitting light signals through waveguides is an effective approach to amplify *R*. Despite the relatively low *R*, all‐optical logical operations can be achieved in existing silicon photonics processes by increasing the optical power. Figure S7 (Supporting Information) shows the variation of 532 and 1550 nm responsivities with MoS_2_ thickness. The results reveal a positive correlation between *R* and the thickness of MoS_2_ at 532 nm, and the light absorption efficiency is higher in thicker materials. Conversely, the *R* at 1550 nm exhibits a negative correlation with MoS_2_, which can be attributed to the interlayer Coulomb interaction. As the thickness increases, the influence of electrons confined to the MoS_2_/Ge interface on channel carrier transport diminishes, resulting in reduced responsivity.

In order to accurately evaluate D^*^ parameter, the noise power spectra in the frequency range of 1–10^5^ Hz with different gate biases are measured (Figure 3d). The noise power density comes from the lattice scattering and impurity scattering, and the device obtains the smallest noise at 0 V gate voltage. Figure 3e exhibits the variation of the absolute values of *D* ^*^ as a function of gate voltage under both 532 and 1550 nm illumination, which is calculated according to the Section 2 in supporting information. The maximum *D* ^*^ is obtained under the condition of 0 V gate bias, which is related to the low noise of the device under this condition. *V*_d_ is fixed at 1 V in the test of basic all‐optical logical gates. Considering the coincident regions among those maps, *V*_g_ is selected as −0.5, 0, and 0.5 V to compare the values of *R* and *D* ^*^. Additionally, the temporal response of the JFET will affect the data processing capability of all‐optical logical operation, which is crucial to realize future applications.^[^ ^12^ ^]^ Figure 3c,f shows the temporal responses at different gate biases (*V*_d_ = 1 V, *V*_g_ = −0.5, 0, and 0.5 V) under 532 and 1550 nm illumination, respectively. The changes in rise time (*T*_r_) and fall time (*T*_f_) are not noticeable with the gate voltage, and the maximum difference is within four times. To ensure the reliability of the result, the time response stability was proven through multiple tests and is shown in Figure S6 (Supporting Information). The µs level optical response is the basis for achieving fast all‐optical logic operations. Table S1 (Supporting Information) compared time response of our device with other literatures of 2D materials, and our MoS_2_/Ge JFET shows relatively high performance. Table S2 (Supporting Information) lists the values of the aforementioned parameters under *V*_g_ = −0.5, 0, and 0.5 V conditions. The most important parameters in the all‐optical logic operation are *R* and *T*, and *V*_g_ = 0.5 V is selected as the test condition for achieving the basic logical gate through comprehensive consideration.

**Figure 4:**
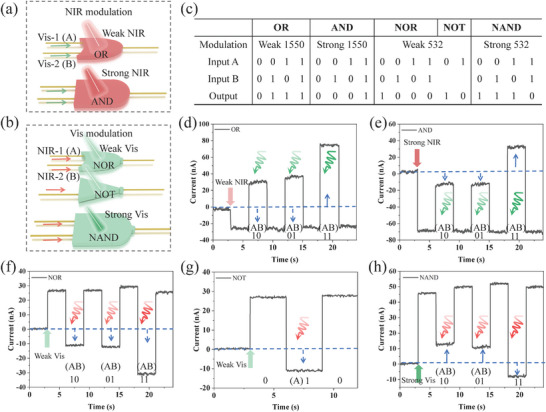
Basically logic gates. a) Schematic diagram of “OR” and “AND” logic implementation under NIR modulation and Vis input conditions. b) Schematic diagram of “NOR,” “NOT,” and “NAND” logic implementation under Vis modulation and NIR input conditions. c) Truth table of the abovementioned five logic gates. Optical current output of “OR” d) “AND” e) logical gates under Vis input of 6.1 µW and NIR modulation of 27.6 µW and 2.9 mW, respectively. The optical current output of “NOR” (f) “NOT” (g) “NAND” (h) logical gates under NIR input of 0.2 mW and Vis modulation of 2.3, 2.3, and 3.8 µW, respectively. In the test of basic logical gates, *V*_d_ and *V*_g_ are 1 and 0.5 V, respectively.

Based on the bipolar photoresponse of the MoS_2_/Ge JFET, five basic logical gates of “OR,” “AND,” “NOR,” “NOT,” and “NAND” are realized on the same device by adjusting the wavelength and power of the modulated and input optics (**Figure** **4**a,b). During logical function verification, the two input signals of optics are defined as A and B, where “1” and “0” represent the on and off states of the optic, respectively. The output signal is electrical, in which “1” and “0” indicate current values larger than or equal to and less than the dark current (reference line in Figure 4d–h), respectively. Figure 4d,e shows the time‐resolved “OR” and “AND” photocurrent curves, respectively. Under the modulation of a weak NIR optic (27.6 µW), there are two Vis input signals. As long as one of the Vis input signals (6.1 µW) is “1”, the output current is above the reference line (0 nA), indicating an output logical state “1”; only when both Vis input signals are simultaneously “0”, the drain current expressed as logic “0”, thus executing the logical “OR” gate. Increasing the power of the modulation NIR optic to 2.9 mW, the photocurrents of the “10” and “01” Vis optic inputs are pulled below the reference line, outputting signal “0”. The output is “1” only when both Vis optics are turned on, which realizes the “AND” logical gate. Different from the traditional electrical design, this structure can achieve the bidirectional transformation between “OR” and “AND” simply by changing the power of the modulation optic, which demonstrates the potential for the construction of complex circuits.

In addition, this structure can realize “NOR,” “NOT,” and “NAND” logic gates using 1550 nm optic input under 532 nm optic modulation, as shown in Figure 4f–h, respectively. Under weak Vis optic modulation (2.3 µW), two NIR input optics of “10,” “01,” and “11” (0.2 mW) can decrease the output currents below the reference line, outputting signal “0”. Meanwhile, the output logic state is “1” when the two NIR input optics are “00”, realizing the logical function of “NOR” or “NOT”. With the power of the Vis modulate optic increased to 3.8 µW, the value of the modulation optic positive photocurrent is between the “10” (“01”) and “11” NIR input negative photocurrent, which executes a “NAND” logical gate. Figure 4c shows a truth table of the five all‐optical logic gates mentioned above, which is consistent with the output of the electrical logic gate.

The all‐optical logic operation of devices holds promising potential in the field of image processing. Taking “AND” logic function as an example, a strong NIR optic and two Vis optics as the modulation and operation signals, respectively (Figure 4e). The remaining image processing functions can be achieved by modifying the modulated and input signals, referring to the implementation methods in Figure 4d–h.

**Figure 5:**
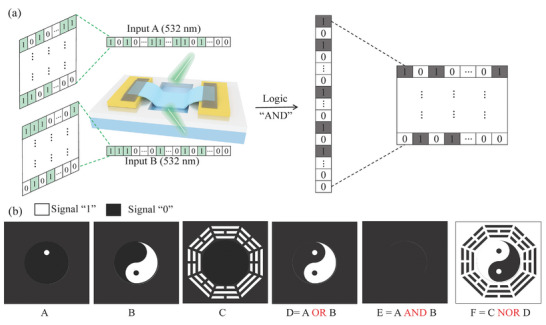
Image processing task. a) Schematic diagram of the “AND” image processing device operation. b) various logical operation results of the input pixel images. White and black represent logical state “1” and “0”, respectively.

As illustrated in **Figure** **5**a, all optical logic realizes black‐and‐white image processing, where black and white correspond to logical states “0” and “1”. First, the color information of each pixel in two M × N pixel images is encoded into binary form following the aforementioned coding rules. Then, These binary matrices are arranged in rows into two 1 × MN matrices, serving as the optical input signals for the device. Through the modulation of strong NIR, the Vis signals with the same index number in the two series undergo logical “AND” operations, and the output results are arranged successively into MN × 1 series. This sequence is subsequently converted back into an M × N matrix. Finally, the “0” and “1” values are decoded, revealing the corresponding color information, and completing the image processing functionality for the two M × N matrices. To validate the feasibility of the logical operations, three‐pixel images are encoded as optics input signals and drain current as output signal for image processing. As shown in Figure 5b, a complete Tai Chi Ba Gua pixel diagram is obtained through various image operations.

**Figure 6:**
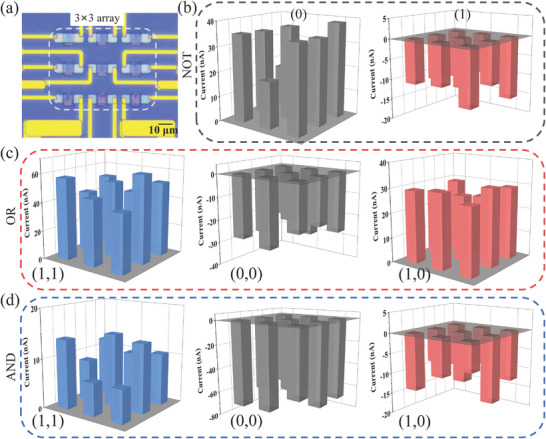
Logic gates on an array. a) Optical microscope image of a 3 × 3 MoS_2_/Ge JFET array. 3D histograms of the output current representing the logical gates of NOT b), OR c), AND d) implemented within the array.

The development of 2D material‐related devices has gradually focused on the reliability of devices and the integration of chips.^[^ ^25^ ^]^ To realize the expansion of all‐optical logic discrete devices into large‐scale integrated circuits, a 3 × 3 array of MoS_2_/Ge JFET devices (**Figure** **6**a) is successfully fabricated. Each unit within the array performs the aforementioned five logical states, thereby verifying the stability of the array. The output current states under the input conditions of (0,0)/(0), (1,0)/(1), and (1,1) are represented by black, red, and green, respectively. As illustrated in Figure 6b–d and Figure S8 (Supporting Information), all nine units within the array effectively achieve the logical gates of “NOT,” “OR,” “AND,” “NOR,” and “NAND,” consistent with the results in Figure 4d–h. The results presented in this study demonstrate the stability, low cost, and high yield characteristics of MoS_2_/Ge JFET, offering possibilities for designing silicon‐based integrated circuits. However, the interface stability of heterojunctions and metal Ohmic connections still presents some challenges to be addressed in the next steps.^[^ ^26^ ^]^

**Figure 7:**
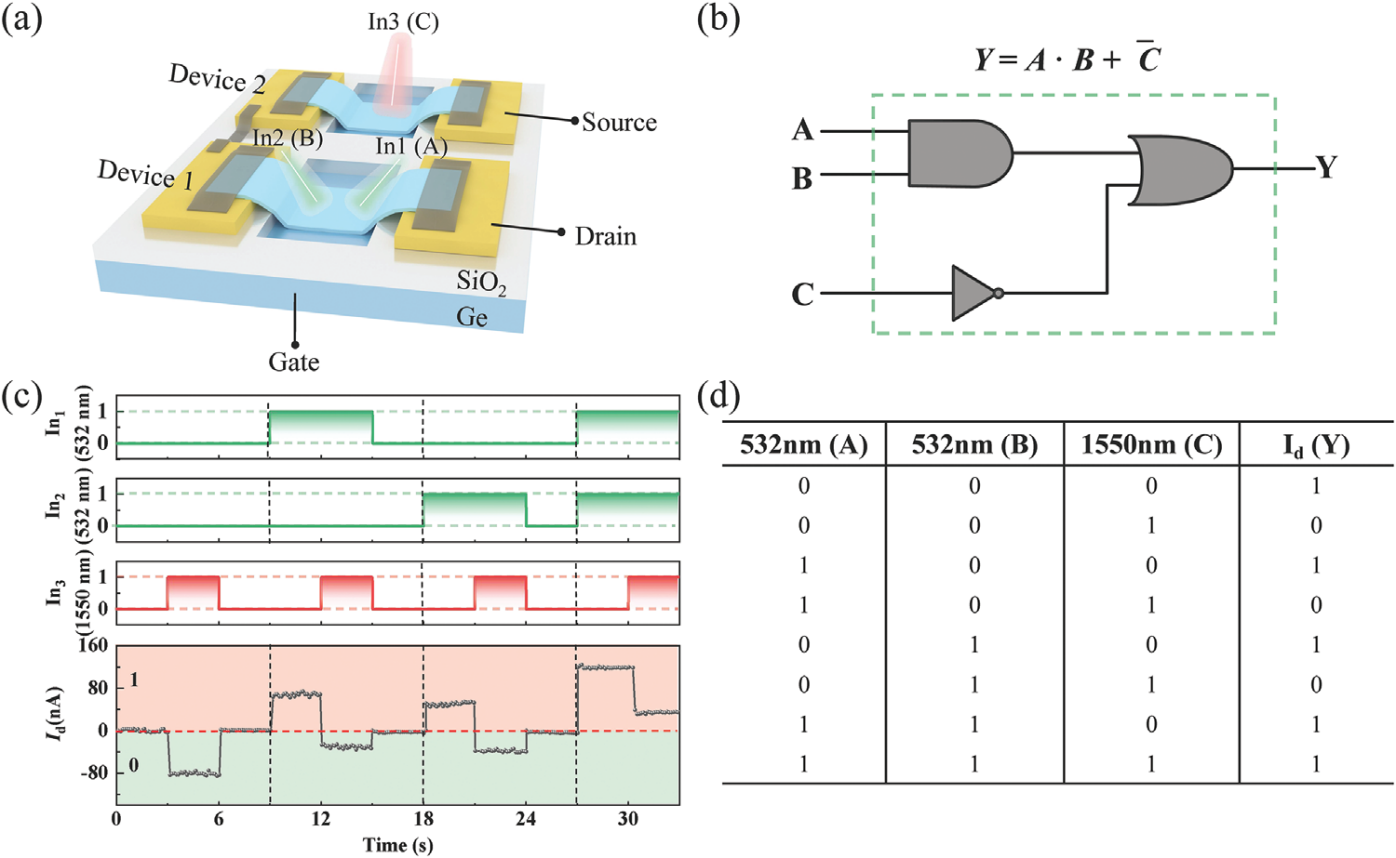
Complex logic gate. a) Schematic illustration of the all‐optical logical circuit connected in series with two MoS_2_/Ge JFET devices. The powers of the Vis and NIR optics are 3.5 and 12.5 µW, respectively. b) Schematic of the logic gate circuit of *Y* = *A*⋅*B* + $\bar{C}$⋅c) Dynamic photoresponse characteristics of the structure for different input logic states as a function of time. d) Output logical truth table under the condition of different optic input combinations. In this case, the output logic state “1” is defined as the value of current larger or equal to the dark current, while the logical state “0” is defined as the value of current less than the dark current.

Building on the implementation of five basic all‐optical logic gates on the same device, more complex logic operations can be realized by integrating two MoS_2_/Ge JFETs in series. An optical microscope image is displayed in Figure S9 (Supporting Information). As shown in **Figure** **7**a, two weak Vis optics and one weak NIR optic are used as input optics on devices 1 and 2, respectively. The logic operation of “*Y* = *A*·*B* + $\bar{C}$ ” is targeted and achieved by adjusting the power matching of the three input signals (In_1_, In_2_ = 3.5 µW, In_3_ = 12.5 µW), and the corresponding logical circuit is displayed in Figure 7b. Furthermore, the time‐resolved output photocurrent measurements are recorded at different input optics (Figure 7c). In this configuration, the “0” and “1” logical gates of optical input and current output are consistent with the previous definition. The photocurrent of the device is ≈−80 nA with only the NIR optic (In_3_) as the input signal‐“001”, outputting signal “0”. This value is ≈+60 nA with one of Vis optics (In_1_ or In_2_) input signal “010” or “100”, outputting signal “1”. When the NIR optic is turned off and the logical gate of two Vis optics are “1” at the same time (input signal “110”), the photocurrent of the device is ≈+118 nA, outputting signal “1”. The negative photocurrent value of the NIR optics (In_3_) is between the positive photocurrents of a single Vis optic (In_1_ or In_2_) and two Vis optics (In_1_ and In_2_). Under NIR optic (In_3_) illumination on device 2, if either Vis input is true (In_1_, In_2_ = “01” or “10”) or both are false (“00”) on device 1, the output values are below the reference line and represent logic “0”. Excluding the above combination of input optics, the output logic is “1” in other optics illumination conditions. Figure 7d shows the truth table of electrical outputs in response to the optics input, and the designed logical operation of “*Y* = *A*·*B* + $\bar{C}$ ” is achieved. In addition, other logic operation functions are also realized in this structure by changing the wavelength and power on devices 1 and 2, respectively. As shown in Figure S10a (Supporting Information), two weak NIR optics (In_1_, In_2_) and one weak Vis optic (In_3_) are inputted on devices 1 and 2, respectively. Figure S10b (Supporting Information) represents the design and implemented logical operation of “*Y* = $\bar{A}$·$\bar{B}+\bar{B}$·*C* + $\bar{A}$·$\bar{C}$” at appropriate power levels (In_1_, In_2_ = 4.2 µW, In_3_ = 12.5 µW). In this condition, the Vis positive (input signal “001”) photocurrent value is between single (input signal “100” or “010”) and two NIR negative (input signal “110”) photoresponses. Figure S10d (Supporting Information) shows the truth table under different optic input combinations. When the inputs of In_1_, In_2,_ and In_3_ are “00110011”, “00001111”, and “01010101”, respectively, the output electrical logic gate is “11010100”. Therefore, a complex logical operation function of “*Y* = $\bar{A}$·$\bar{B}+\bar{B}$·*C* + $\bar{A}$·$\bar{C}$” is realized by integrating two MoS_2_/Ge JFETs. Based on this logical operation, the input of the Vis optic (In_3_) on device 2 is increased to 6.1 µW, so that both the output values under illumination of one (input signal “100” or “010”) and two (input signal “110”) NIR optics are less than that of a single Vis optic (input signal “001”), realizing the logical function of “*Y* = $\bar{A}$·$\bar{B}$ + *C*” (Figure S11, Supporting Information).

**Figure 8:**
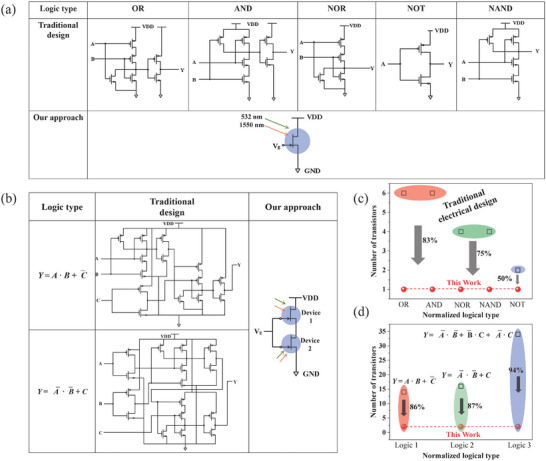
Circuit or optical diagrams. Comparison of the circuit diagrams a) and the transistor numbers c) of “OR,” “AND,” “NOR,” “NOT,” and “NAND” between the traditional design and the all‐optical device. Comparison of the circuit diagrams b) and the transistor number d) of complex logical operations between the traditional design and the all‐optical device.

Furthermore, the all‐optical logical operations based on these designs demonstrate significant advantages in reducing the transistor number and increasing circuit integration. **Figure** **8**a compares the circuit diagrams between all‐optical logical operations and traditional electrical designs for implementing “OR,” “AND,” “NOR,” “NOT,” and “NAND” logic gates. In traditional designs, 6, 6, 4, 2, and 4 transistors are required to realize the abovementioned logical gates. In contrast, the present approach only requires one transistor, with these five basic logic gates implemented by adjusting the wavelength and power of the input optics. As shown in Figure 8c, the transistor number of this work is reduced by 83% for (“OR”, “AND”), 75% for (“NOR”, “NAND”), and 50% for “NOT” gates compared to traditional circuits. For complex logical operations, the benefit of all‐optical in terms of improving integration is further amplified. Figure 8b shows the logic operations of “*Y* = *A*·*B +* $\bar{C}$” and “*Y* = $\bar{A}$·$\bar{B}$ + *C*” are implemented using traditional circuit connections, which require 14 and 16 transistors, respectively. Figure S12 (Supporting Information) shows the electrical logic circuit diagram for 34 transistors to implement the “*Y* = $\bar{A}$·$\bar{B}+\bar{B}$· *C* + $\bar{A}$·$\bar{C}$” function. However, all‐optical logical operations only require two transistors to perform these complex logical functions by modulating the input optics. Compared to electrical implementation methods, this work consumes the smallest number of transistors, with reductions of 86%, 87%, and 94% for “*Y* = *A*·*B +* $\bar{C}$ *”*, “*Y* = $\bar{A}$·$\bar{B}$ + *C*”, and “*Y* = $\bar{A}$·$\bar{B}+\bar{B}$·*C* + $\bar{A}$·$\bar{C}$”, respectively (Figure 8d). These all‐optic logical operations present a new strategy for signal computing, communication, and processing, with the potential to revolutionize next‐generation photonics technology and enable more applications.

## 3 Discussion

In this work, we have designed and fabricated a MoS_2_/Ge JFET with a wavelength‐dependent bidirectional photoresponse that originates from the competition between photoconductivity and interlayer Coulomb interactions in the material. By using different wavelengths (Vis or NIR optic) and powers (weak or strong optic intensity) as combinations of modulation and input signals, the five logic gates of “AND,” “OR,” “NAND,” “NOT,” and “NOR” were implemented on a single device within a 3 × 3 array, and validates the feasibility and potential in the field of image processing. In addition, more complex all‐optical logical operations were achieved by integrating two MoS_2_/Ge JFETs in series. Compared to traditional electrical designs for constructing circuits, our approach reduces the number of transistors by 83%, 83%, 75%, 75%, 50%, 86%, 87%, and 94% for implementing the logical operations of “AND,” “OR,” “NAND,” “NOT,” “NOR,” “*Y* = *A*·*B +* $\bar{C}$,” *Y* = $\bar{A}$·$\bar{B}+\bar{B}$·*C* + $\bar{A}$·$\bar{C}$“ and ”*Y* = $\bar{A}$·$\bar{B}$ + *C*,″ respectively. These results demonstrate the transformative potential of all‐optical logic operations in the realm of photonics, heralding a new technological paradigm for rapid and precise processing of optical signals.

## 4 Experimental Section

### Device Fabrication

The MoS_2_/Ge JFET was fabricated on a patterned substrate. First, a 100 nm thick SiO_2_ layer was deposited on the Ge surface by chemical vapor deposition as an insulating layer. Second, the Ge windows were patterned by photolithography and reactive ion etching for contact with the gate electrodes. Third, Ni/Au (5/100 nm) were deposited as a metal electrode using e‐beam evaporation, followed by lift‐off in acetone. Finally, MoS_2_ and graphite were mechanically exfoliated from crystals and transferred to the substrate using a microtransfer manipulation platform.

The fabrication processes for the 3 × 3 MoS_2_/Ge JFET array remained consistent with those of individual devices, with slight variations in the materials. Multilayer MoS_2_ was selected from commercially available 1 cm × 1 cm materials purchased from Hex Carbon Technologies, while the contact electrode material was replaced with Au instead of graphite.

### Material Characterization and Measurements

The thicknesses of the 2D materials were measured with an AFM (Asylum Research Cypher S), which was equipped with Adama Conductive diamond‐coated Si tips (FM‐LC). The Raman spectra were analyzed using a Lab Ram HR800 with a 532‐nm excitation laser. Furthermore, the contact potential difference between MoS_2_ and Ge was measured using KPFM (Ntegra Prima).

The electronic and photoelectronic characteristics of the devices were investigated using a Keithley 4200‐SCS semiconductor parameter analyzer under both dark conditions and 532/1550 nm optical illumination. Additionally, a tungsten halogen lamp (0.3–2.5 µm, GloriaT250A, Zolix instruments) was employed as a broadband light source during the response spectra measurements. Prior to illuminating the device, the broadband light was monochromatized using a monochromator (Omni‐λ300i, Zolix Instruments) and coupled into a multimode optical fiber.

## Conflict of Interest

The authors declare no conflict of interest.

## Author Contributions

J.Y. was responsible for project management and manuscript preparation. Z.H., N.Z., Q.Z., Y.Z., Y.L., and Y.L. were in charge of background investigation and data curation. J.A., Z.J., Z.Z., and H.G. provided guidance in conceptualization and methodology. H.H., L.W., and Z.Z. supervised the project.

## Supporting information

Supporting Information

## Data Availability

The data that support the findings of this study are available from the corresponding author upon reasonable request.
