# Supplementary material for: All‐Optic Logical Operations Based on the Visible‐Near Infrared Bipolar Optical Response
Source: Adv Sci (Weinh). 2024 Jul 23;11(40):2404336. doi: 10.1002/advs.202404336 (PMC11516116; doi:10.1002/advs.202404336)
Supplement: Supplementary file 1 — Supporting Information [file ADVS-11-2404336-s001.docx]

All-optic logical operations based on the visible-near infrared bipolar optical response

Jie You^1,2^, Zhao Han^1^, Ningning Zhang^1^, Qiancui Zhang^1^, Yichi Zhang^1^, Yang Liu^1^, Yang Li^2^, Jinping Ao^2^, Zuimin Jiang^3^, Zhenyang Zhong^3^, Hui Guo^1^, Huiyong Hu^1^, Liming Wang^1,*^ Zhangming Zhu^1,*^

^1^ Key Laboratory of Analog Integrated Circuits and Systems (Ministry of Education), School of Integrated Circuits, Xidian University, Xi’an, 710071, China

^2^ School of Integrated Circuits, Jiangnan University, Wuxi, Jiangsu 214000, China

^3^ State Key Laboratory of Surface Physics, Department of Physics, Fudan University, Shanghai 200433, China

**Section 1: Supporting figures and tables.**


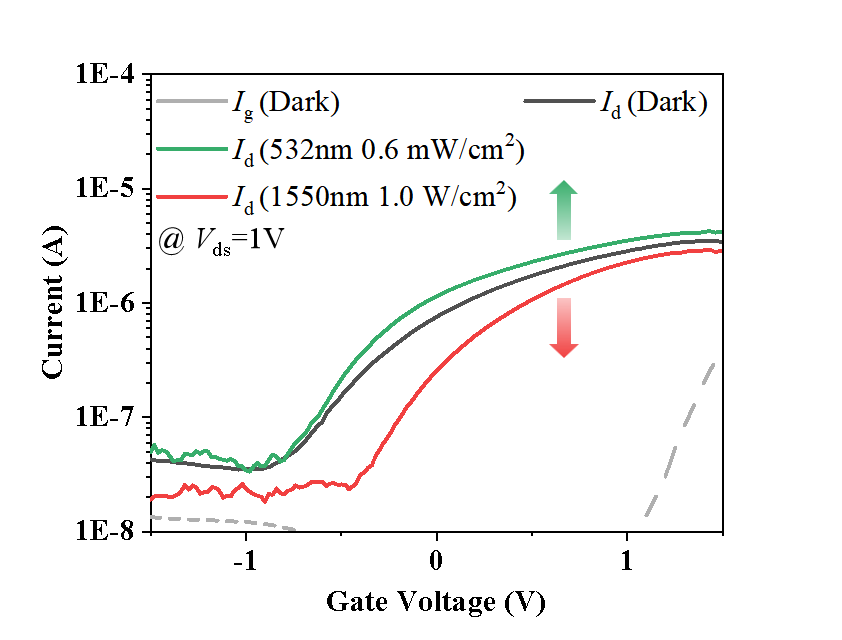


**Fig. S1.** **Transfer characteristic**. Log-scaled transfer curves of the structure under dark and optical illumination.


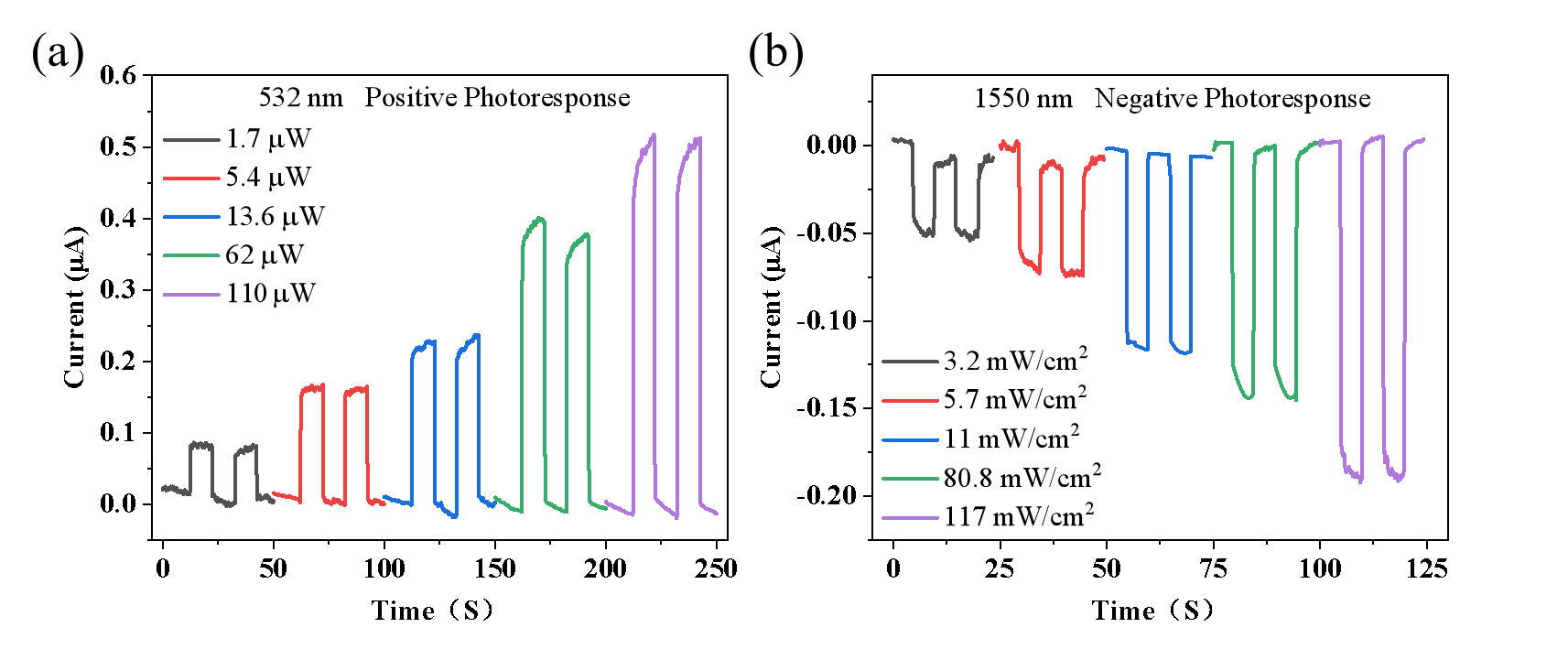


**Fig. S2.** **Positive and negative photo response.** Dynamic photo response of the MoS_2_/Ge JFET with different power under 532 (a) and 1550 nm (b) illumination.


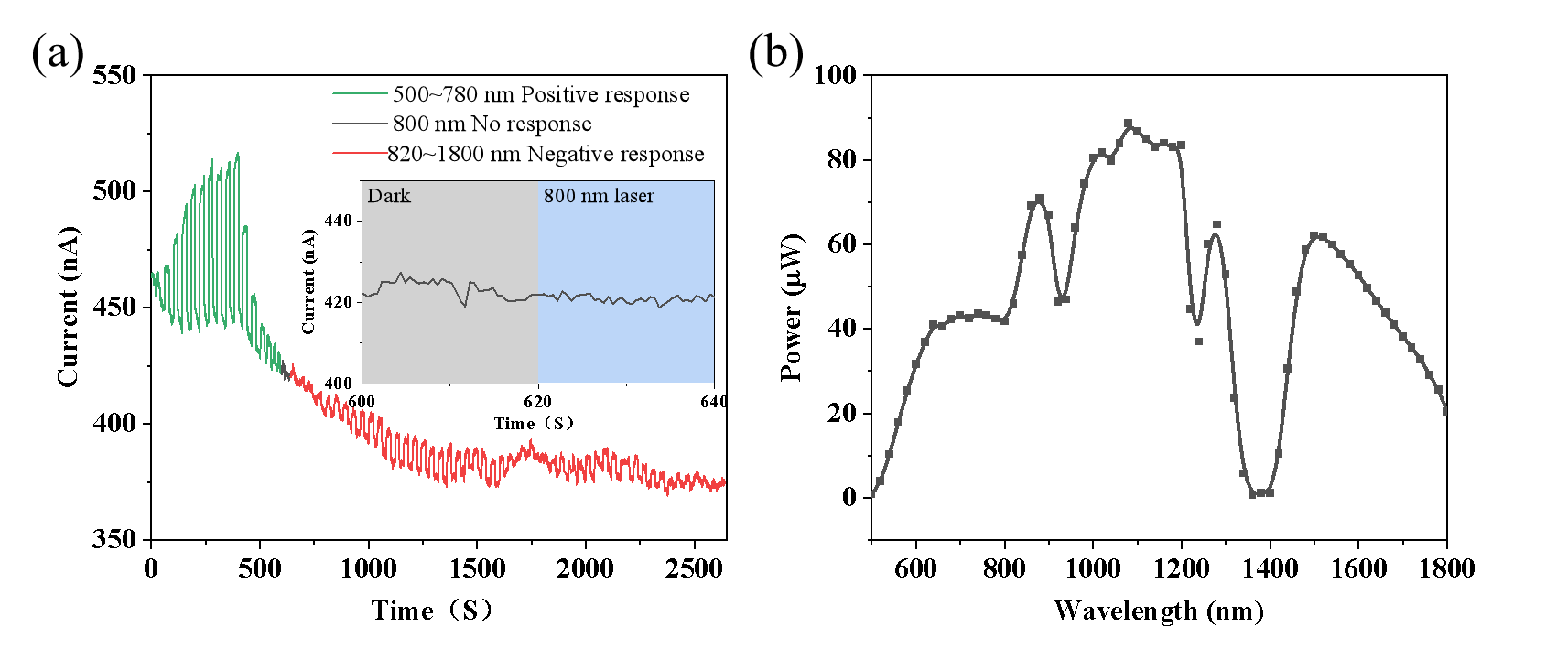


**Fig. S3. Response spectra.** (a) Dynamic photoresponse of the MoS_2_/Ge JFET in the range of 500-1800 nm. (b) The optical power of tungsten halogen lamp at different wavelengths.

Fig. S3a shows the dynamic optical response of the device in the range of 500-1800nm. Notably, the device exhibits positive photoresponse within the range of 500-780 nm, while shows negative photoresponse within the range of 820-1800 nm. The depiction in Fig. S3a specifically illustrates the optical response of the device at 800 nm, where the net photocurrent approaches zero. Figure S3b shows the power values of the lamp at various wavelengths measured by the optical power meter.


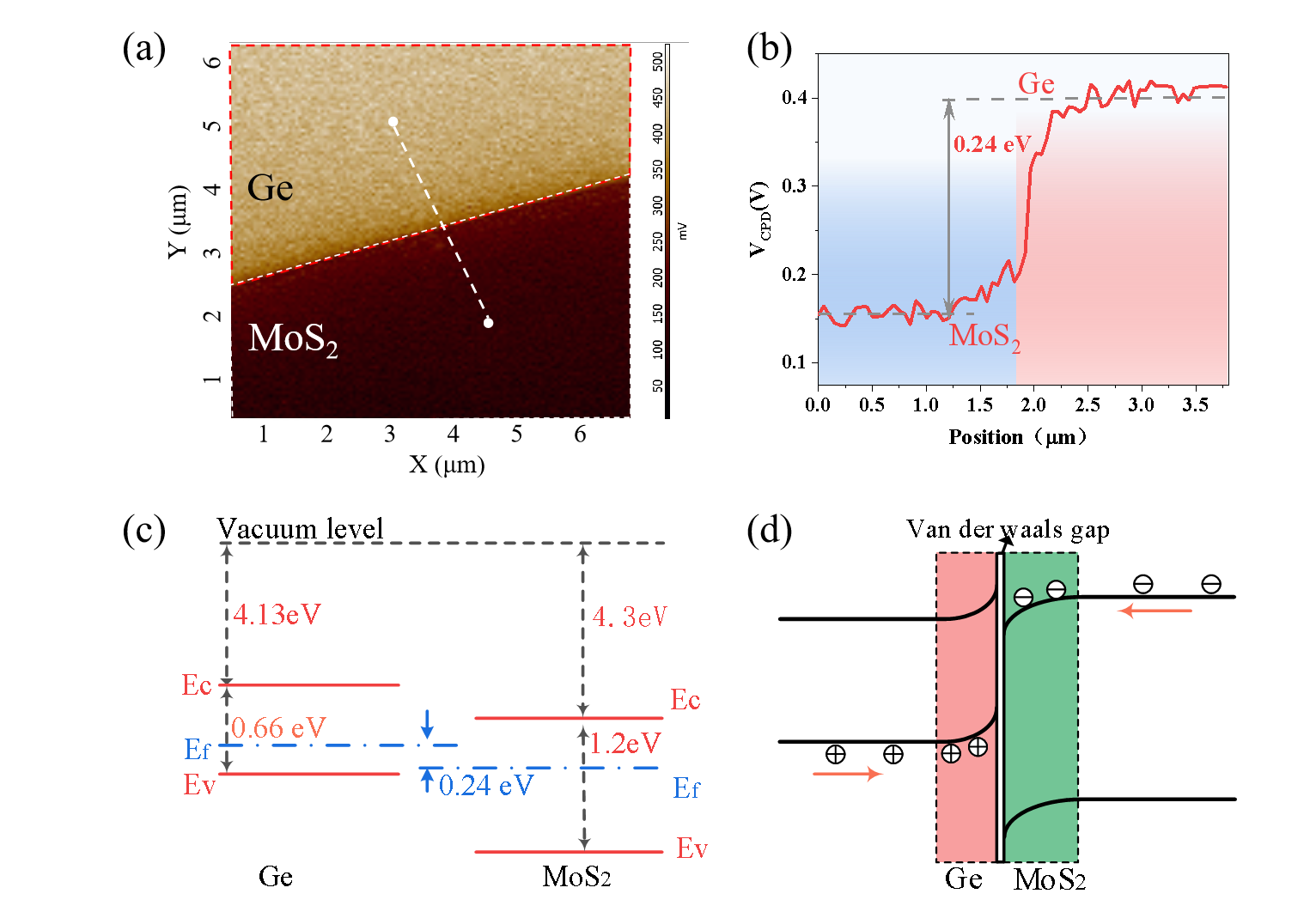


Fig. S4. Energy band diagrams. (a) KPFM image obtained from MoS_2_-Ge heterostructure, in which MoS_2_ is on the Ge substrate. (b) KPFM result taken along the white line in (a). Energy band diagrams of MoS_2_ and Ge before (c) and after (d) they are in contact, respectively.

**Fig. S5. PL spectrum.** PL spectrum of the MoS_2_/Ge JFET measured at 77K.


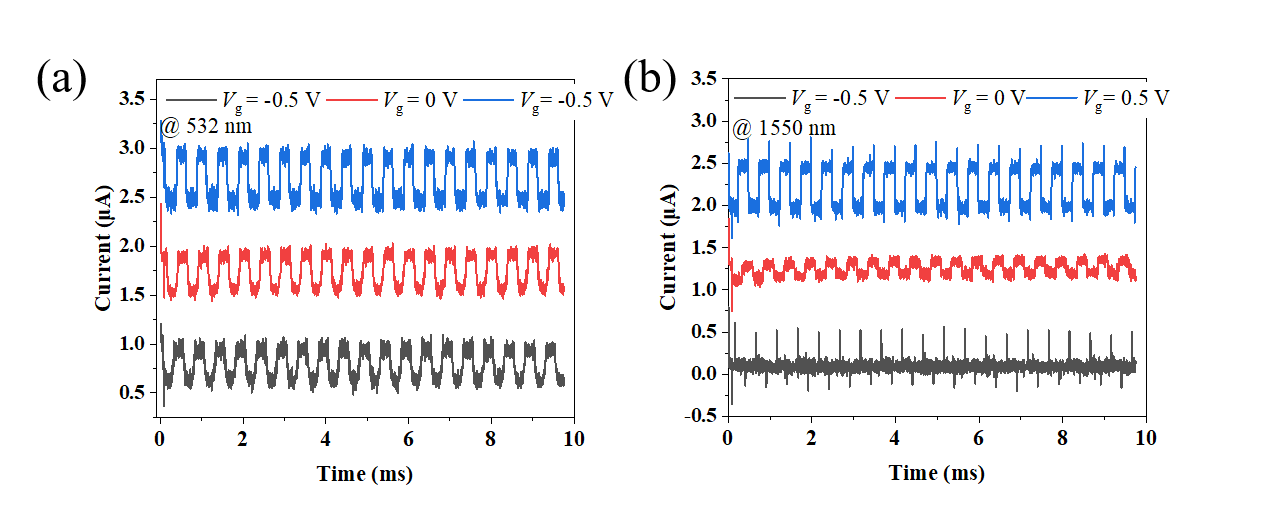


Fig. S6. Switching characteristics. Periodic time response of the JFET at *V*_g_ = -0.5 V, 0 V and 0.5 V conditions under illumination of 532 (a) and 1550 nm (b), respectively.


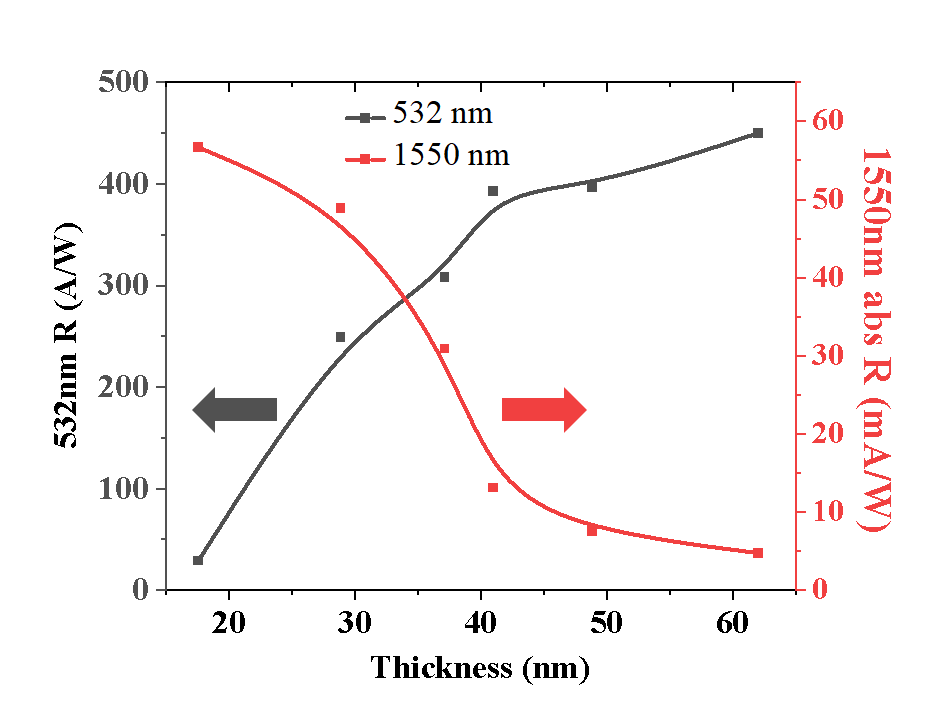


**Fig. S7.** **Responsiveness varies with thickness.** Responsivities under 532 nm and 1550 nm illumination as a function of MoS_2_ thickness

**Table S1.** **Comparison of response time**.The response time of MoS_2_/Ge JFET and other literatures

| Structure | Wavelength(nm) | Response time(μs) | ref |
| --- | --- | --- | --- |
| WSe_2_/ZnO | 637 | 10 | [1] |
| ReS_2_/MoTe_2_ | 532 | 109 | [2] |
| WSe_2_ | 520 | 16 | [3] |
| MoS_2_/ZnO | 532 | 0.9s | [4] |
| MoS_2_/CuPc | 500 | 436 | [5] |
| MoS_2_/Ge JFET | 532 | 42 | This Work |

Table S2. Comparison of parameters. The statistical parameters values of *R*, *D*^*^, *T*_r_, and *T*_f_ under the condition of *V*_g_ = -0.5, 0 and 0.5V at 532 and 1550 nm illumination.

|  | ***R*** **(A/W)** | | ***D*^*^ (Jones)** | | ***T*_r_ (μs)** | | ***T*_f_ (μs)** | |
| --- | --- | --- | --- | --- | --- | --- | --- | --- |
|  | 532 nm | 1550 nm | 532 nm | 1550 nm | 532 nm | 1550 nm | 532 nm | 1550 nm |
| *V*_g_ = -0.5V | 20.8 | -0.002 | 1.8×10^10^ | 1.4×10^6^ | 65 | 25 | 50 | 7.5 |
| *V*_g_ = 0V | 112.4 | -0.03 | 1.1×10^11^ | 2.3×10^7^ | 37.5 | 19.2 | 42.5 | 10 |
| *V*_g_ = 0.5V | 147.2 | -0.03 | 1.2×10^11^ | 1.9×10^7^ | 12.5 | 17.5 | 45 | 7.5 |


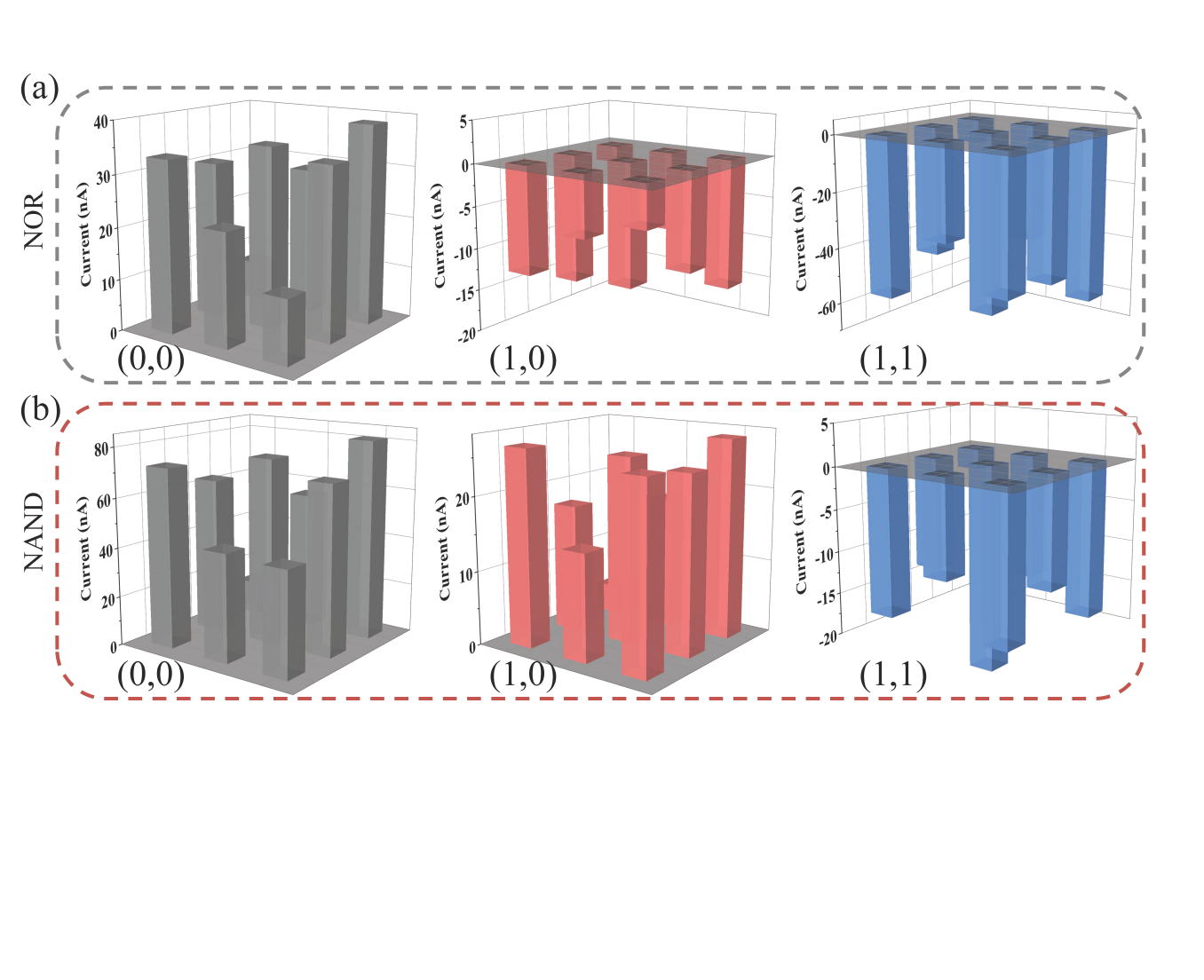


**Fig. S8. Basically logic gates on a array.** Three-dimensional histograms of the output current representing the logical gates of NOR (a) and NAND (b) implemented within the array.


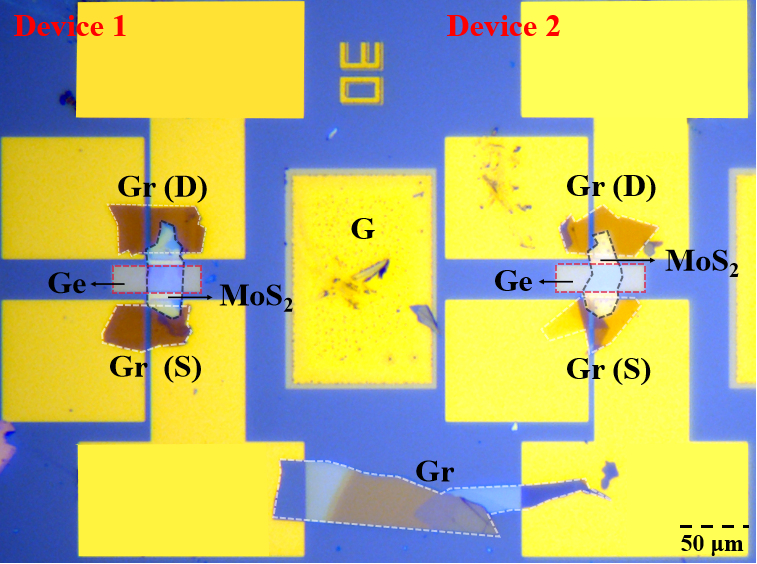


Fig. S9. Device structure. Optical microscope image of the structure with two MoS_2_/Ge JFETs in series.


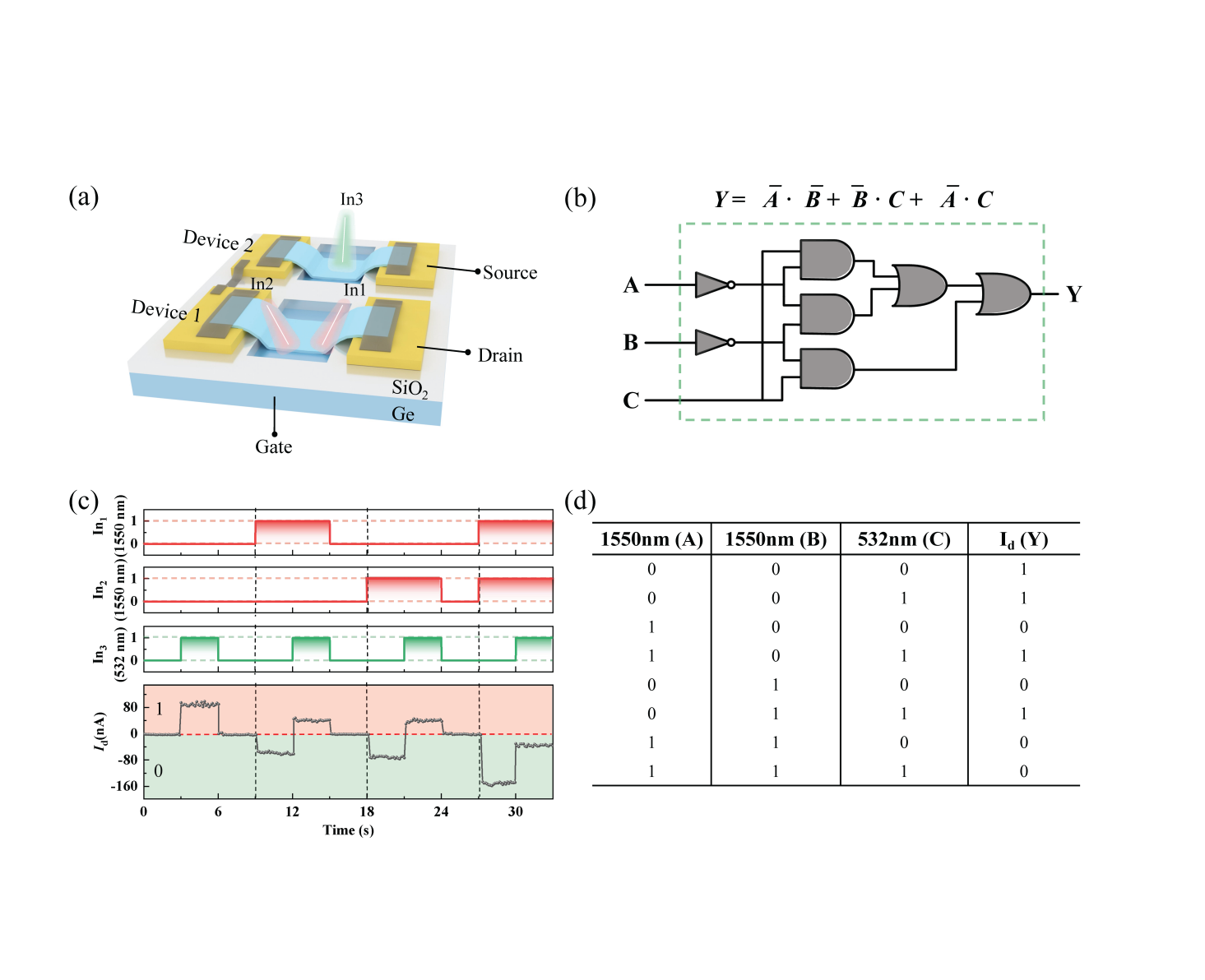


Fig. S10. Complex logic gate. (a) Schematic illustration of the all-optical logical circuit connected in series with two MoS_2_/Ge JFET devices. The power of visible and infrared optics are 4.2 and 12.5 μW, respectively (b) Logic operation function schematic circuit of “*Y* = ‾*A*·‾*B* + ‾*B*· *C* + ‾*A*·‾*C*” .(c) Dynamic photoresponse characteristics of the structure for different input logic states as a function of time. (d) Output logical truth table under the condition of different optic input combinations.


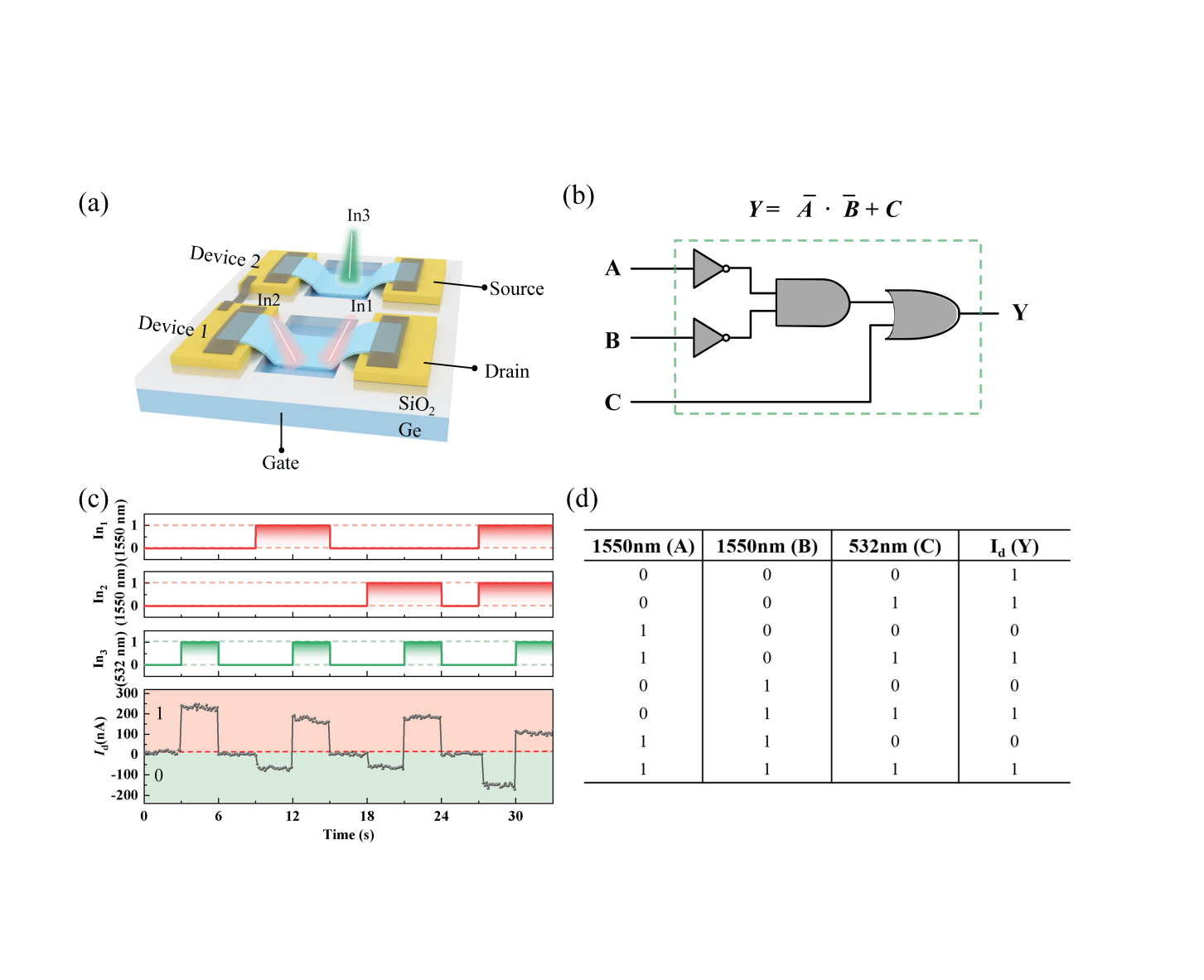


Fig. S11. Complex logic gate. (a) Schematic illustration of the all-optical logical circuit connected in series with two MoS_2_/Ge JFET devices. The power of visible and infrared optics are 6.1 and 12.5 μW, respectively. (b) Logic operation function schematic circuit of “*Y* = *‾A ‾B* + *C*”. (c) Dynamic photoresponse characteristics of the structure for different input logic states as a function of time. (d) Output logical truth table under the condition of different optic input combinations.

Fig. S12. Circuit diagram. Electrical logic circuit diagram of “*Y* = ‾*A* · ‾*B* + ‾*B* · *C*+ ‾*A* · *C*”.

**Section 2: *D*^*^ Calculation method.**

The total square noise current (<in^2^>) can be determined by integrating the noise power density,

where S_n_($f$) is the noise power density, B is the maximum bandwidth.

In addition, the noise equivalent power (NEP) can be calculated by the following formula:

Where *R* is the responsivity of the photodetector. The normalized *D*^*^ can be determined by:

$$D^{*}=\frac{\sqrt{A}\sqrt{B}}{NEP}$$

Where A is the area of the device.

Referrence：

[1] N. Guo, L. Xiao, F. Gong, M. Luo, F. Wang, Y. Jia, H. Chang, J. Liu, Q. Li, Y. Wu, Y. Wang, C. Shan, Y. Xu, P. Zhou, W. Hu, Adv Sci (Weinh) 2020, 7 (1), 1901637, <https://doi.org/10.1002/advs.201901637>.

[2] M. Luo, X. Chen, P. Wu, H. Wang, Y. Chen, F. Chen, L. Zhang, X. Chen, Optical and Quantum Electronics 2019, 51 (5), <https://doi.org/10.1007/s11082-019-1839-3>.

[3] Y. Tang, Z. Wang, P. Wang, F. Wu, Y. Wang, Y. Chen, H. Wang, M. Peng, C. Shan, Z. Zhu, S. Qin, W. Hu, Small 2019, 15 (12), e1805545, <https://doi.org/10.1002/smll.201805545>.

[4] J. Zhang, Y. Liu, X. Zhang, Z. Ma, J. Li, C. Zhang, A. Shaikenova, B. Renat, B. Liu, ChemistrySelect 2020, 5 (11), 3438, <https://doi.org/10.1002/slct.202000746>.

[5] Z. H. Xu, L. Tang, S. W. Zhang, J. Z. Li, B. L. Liu, S. C. Zhao, C. J. Yu, G. D. Wei, Materials Today Physics 2020, 15, <https://doi.org/10.1016/j.mtphys.2020.100273>.
